# Antileishmanial Drug Discovery and Development: Time to Reset the Model?

**DOI:** 10.3390/microorganisms9122500

**Published:** 2021-12-02

**Authors:** Ana Isabel Olías-Molero, Concepción de la Fuente, Montserrat Cuquerella, Juan J. Torrado, José M. Alunda

**Affiliations:** 1 Department of Animal Health, Faculty of Veterinary Medicine, Universidad Complutense de Madrid, 28040 Madrid, Spain; anaolias@ucm.es (A.I.O.-M.); cfuente2@ucm.es (C.d.l.F.); mcayensa@ucm.es (M.C.); 2 Department of Pharmaceutics and Food Technology, Faculty of Pharmacy, Universidad Complutense de Madrid, 28040 Madrid, Spain; torrado1@ucm.es

**Keywords:** leishmaniasis, visceral leishmaniasis, *Leishmania*, chemotherapy, drug discovery, drug pipeline, surrogate models, in vitro, in vivo, in silico

## Abstract

Leishmaniasis is a vector-borne parasitic disease caused by *Leishmania* species. The disease affects humans and animals, particularly dogs, provoking cutaneous, mucocutaneous, or visceral processes depending on the *Leishmania* sp. and the host immune response. No vaccine for humans is available, and the control relies mainly on chemotherapy. However, currently used drugs are old, some are toxic, and the safer presentations are largely unaffordable by the most severely affected human populations. Moreover, its efficacy has shortcomings, and it has been challenged by the growing reports of resistance and therapeutic failure. This manuscript presents an overview of the currently used drugs, the prevailing model to develop new antileishmanial drugs and its low efficiency, and the impact of deconstruction of the drug pipeline on the high failure rate of potential drugs. To improve the predictive value of preclinical research in the chemotherapy of leishmaniasis, several proposals are presented to circumvent critical hurdles—namely, lack of common goals of collaborative research, particularly in public–private partnership; fragmented efforts; use of inadequate surrogate models, especially for in vivo trials; shortcomings of target product profile (TPP) guides.

## 1. Chemotherapy of Leishmaniasis: Current Drugs, Limitations, and Prospective Treatments

Leishmaniasis is a group of vectorial parasitic diseases widely distributed in the world. The etiological agent is a protozoon of the genus *Leishmania* (Kinetoplastida, Trypanosomatidae) with a digenetic cycle involving a vertebrate host and a vector sand fly of the genera *Phlebotomus* or *Lutzomyia* (Diptera, Psychodidae). Leishmaniasis is considered a neglected tropical disease (NTD), present in a total of 98 countries although between 70 and 90% of human cases are diagnosed in 15 countries: Ethiopia, Kenya, Algeria, Sudan, South Sudan, Syria, Afghanistan, Iran, India, Bangladesh, Brazil, Colombia, Costa Rica, Bolivia, and Peru. Estimated annual incidence reaches between 0.9 and 1.7 million cases, and the disease causes 20,000–30,000 deaths per year [1,2]. Moreover, new transmission patterns have caused the emergence of the infection in intravenous drug users [3], blood transfusions [4], and solid organs transplant recipients [5,6,7,8], thus extending the infection to regions previously unaffected [9,10].

Leishmaniasis is a spectral disease, and signs and lesions frequently associated with infection include skin ulcers, mucosal destruction, wasting, recurrent irregular fever, hepatosplenomegaly, and anemia. The appearance and severity of these signs, as well as clinical course and prognosis of the disease, vary depending on the *Leishmania* species involved and the immune status of the host, ranging from asymptomatic cryptic infections to fatal diseases. Three main clinical forms of the disease can be differentiated in humans: cutaneous (CL), mucocutaneous (MC), and visceral leishmaniasis (VL), besides post-kala-azar dermal leishmaniasis (PKDL) [11,12]. VL, although less frequent, is the most severe disease with frequent fatal outcomes unless patients are treated. Two types of VL are recognized, anthroponotic VL, caused by *L. donovani*, which has humans as the main reservoir, and zoonotic VL (ZVL), caused by *L. infantum* and affecting both humans and dogs [13].

No vaccine for human leishmaniases is available, while some vaccines against *L. infantum* in dogs have been registered and marketed. Thus far, there is insufficient information on their efficacy due to the lack of standardization, methodological shortcomings, and substantial differences in the characteristics of studied populations, besides other limitations [14]. Moreover, there are environmental concerns about the use of insecticides to interrupt the transmission cycles. Naturally, infected dogs are considered as the main reservoir for *L. infantum* (=*L. chagasi*) (ZVL) [15], and dog culling has been suggested to reduce the prevalence of the disease in some endemic regions [16,17]. However, its effect in those areas is controversial [18,19,20], and ethical considerations would make this measure unacceptable in other endemic areas (e.g., Western Europe). Under such circumstances, control of leishmaniases, for the most part, relies on chemotherapy.

Google Scholar search treatment of leishmaniasis yielded 290,000 entries (accessed on 22 November 2021) and one of the frequently used academic bibliographic sources, PubMed, recovered over 13,600 published papers (November 2021); these figures show that the topic is well represented in academia. More refined analysis of these numbers shows that, in many cases, the actual relationship of the contributions to the chemotherapy of the disease is low. This occurs in both human and dog leishmaniasis [21].

Around 25 drugs or combinations have been used for humans in leishmaniasis [22,23,24]. This high number suggests that there are limitations in the currently available chemotherapy. Some years ago, the WHO-OMS (2004) concluded that the most promising drugs for the treatment of leishmanial infections were amphotericin B in its liposomal presentation, miltefosine, and paromomycin (https://www.who.int/health-topics/leishmaniasis#tab=tab_1; accessed on 26 November 2021).

Investigation on potentially new drugs and targets has been very active in recent decades, and several reviews have been published [23,25,26,27,28,29,30,31,32,33,34,35,36,37,38,39,40,41,42]. A wide range of compounds and chemical families have been identified as potential hits and leads, and some of them have been or are being tested in clinical trials. Among the most promising candidates are compounds impairing thiol metabolism [31], aminopyrazoles, pyrazolopyrimidines, proteasome inhibitors, nucleoside analogs [38,41], and repurposed compounds (e.g., nitroaromatic compounds, oxaboroles) [38]. In addition, current antileishmanial chemotherapy still relies on pentavalent antimonials, amphotericin B, pentamidine isethionate, miltefosine, and paromomycin [42]. However, these drugs are far from being novelties or providing an ideal chemotherapeutic control of the disease. To cope with the limitations of these drugs, several combinations have been tested and are actually employed in clinical practice [43,44] and less toxic drug delivery systems (DDSs), such as PLGA nanoparticles or liposomes [41], and presentations (e.g., polyaggregated forms of AmB) [45] or amphiphilic antimony [46].

### 1.1. Antimonials

Pentavalent antimonials (Sb^V^) have been available since 1920 and in the form of stibogluconate since 1945. They have been the frontline monotherapy treatment for VL, and they still are in canine leishmaniasis (CanL) [47,48,49,50]. After being extensively employed for 70 years, their mechanism of action (MoA) remains not completely understood, although a variety of biochemical effects have been described (e.g., inhibition of DNA topoisomerase I, interference with the peculiar glutathione of trypanosomatids—trypanothione—and glycolytic enzymes) [51,52,53,54,55,56]. The prevailing view on the MoA relates to the selective intracellular accumulation of Sb^III^ [57,58,59] through the dysfunction of AQP1 transporter, overproduction of thiols, and overexpression of ABC transporters (e.g., LABCI4, MRPA) [60,61,62].

Two main reasons have restricted the use of antimonials—namely, side effects/toxicity and the emergence of resistance and therapeutic failures, notably in India (Bihar district), Bangladesh, and Nepal. Medication with antimonials has been associated with local pain with intramuscular injection, and severe side effects including cardiotoxicity, pancreatitis, hepatotoxicity, and nephrotoxicity [63,64,65,66]. However, the mechanism of toxicity of Sb^V^ compounds involved is not fully elucidated and could be associated with the residual Sb^III^ of the preparations [67]. Actually, antimonials are short lived (i.e., 80–95% of the antimonial is eliminated after 6–9 h) [68]; no correlation between Sb^III^ levels and toxicity has been found [69], which perhaps is related to the analytical method employed, and no clear evidence of renal toxicity in the absence of a previous intraglomerular lesion has been presented in dogs [70] when used at the appropriate dosage [71]. Moreover, reports of therapeutic failures/resistance emergence are mainly limited to certain areas of the world—not necessarily those where the therapeutic pressure on the population has been higher. This suggests that an environmental factor [72], such as the presence of arsenic in drinking water in India [73], may act concomitantly with the low dosage and duration and interruption of the treatment [31].

### 1.2. Amphotericin

Amphotericin B (AmB) is a polyene antibiotic obtained through fermentation of *Streptomyces nodosus* and is a reference treatment for systemic fungal infections (e.g., *Candida*). Its antileishmanial activity was discovered in the 1960s [48]. MoA of the antibiotic has been mainly related to the differential binding to ergosterol from *Leishmania* membranes [74,75]. This MoA and the absence of ergosterol in mammalian cells probably explain its leishmanicidal activity and clinical cure of infected individuals and also the almost negligible reports of leishmanial resistance to this molecule. AmB presents two main shortcomings: toxicity of free AmB, deoxycholate, and the high price of safer presentations. Toxicity of AmB can be reduced by using aggregated states of the molecule, although the most successful developments have been lipidic presentations such as Amphocil^®^, Abelcet^®^, and, particularly, liposomal AmB and Ambisome^®^ [41,45,76,77,78,79]. Liposomal presentation is much safer without losing efficacy against leishmanial infections. However, its high price makes it unaffordable for many patients and countries. Low-price liposomal AmB (Fungisome^®^) and other drug delivery systems (e.g., niosomes, microspheres of albumin, chitosan, nanodisks) could be sustainable solutions for low-income areas of the world [80,81].

### 1.3. Miltefosine

Miltefosine, a derivative of alkylphosphocholine, was discovered and developed in the 1980s as an antineoplastic drug for its ability to induce selective apoptosis in tumor cells [82]. Its antileishmanial activity in vivo was found in the 1990s [83] and has been considered an alternative to chemotherapy for leishmaniasis, with clinical efficacy comparable to AmB [84,85,86]. The main inconvenience of this molecule relates to its long half-life (t½) in the organism (>120 h) and its teratogenicity. This excludes the use of miltefosine during the fertile life of women (or they must be subjected to anticonceptive treatment) [87]. In addition, its long t½ and the possibility of self-medication (it can be administered orally without medical supervision) under less-than-strict conditions could favor the emergence of resistance. In fact, it is relatively easy to generate resistant lines of *L. donovani* under laboratory conditions [88], and loss of clinical efficacy has been reported [89,90,91,92,93,94,95,96,97] and confirmed in the laboratory [98].

### 1.4. Paromomycin

Paromomycin (monomycin, aminosidine) is a low-cost aminoglycoside antibiotic produced by Park Davis (now Pfizer) obtained from *Streptomyces krestomuceticus* and discovered in the 1950s. It is effective against a variety of parasitic agents (*Giardia*, *Entamoeba*) and its antileishmanial activity was reported in the 1990s and confirmed afterward [99,100]. MoA is apparently related to the inhibition of protein synthesis by specific binding to 16S ribosomal RNA [101,102]. Low price and scarce toxicity, the short length of treatment, and efficacy seem to make this antibiotic a frontline treatment for leishmaniasis. Unfortunately, resistances are easily generated if used in monotherapy [103,104,105].

### 1.5. Pentamidine

Pentamidine showed high toxicity (e.g., cardiotoxicity, decrease in blood pressure, irreversible insulin-dependent diabetes mellitus) [31,41] when used against VL. MoA has been related to the accumulation of the drug in the parasite and binding to kinetoplast DNA after its entry through arginine and polyamine transporters [31,41,42]. Given the reported toxicity in VL treatment this compound is mainly used against CL [38,42], and *Leishmania* resistance has been associated with the upregulation of the drug efflux, which results in low levels of the aromatic diamine in the cytosol and mitochondrion [31].

Overall, current chemotherapy of leishmaniasis (Table 1) is not satisfactory in terms of routes of administration and length of treatments, the high price of the most effective and safe drugs (e.g., liposomal AmB), severe side effects (e.g., teratogenicity of miltefosine, gastrointestinal disturbances), and toxicity, besides clinical failures [106,107]. Moreover, reports on the emergence of resistance against both the classical drugs and the more recently introduced molecules are increasing, particularly in the areas where they are most needed [32]. Drugs used currently against leishmaniasis can elicit the clinical cure of infected individuals, but none of the available drugs induces a parasitological cure. This has been observed in human cases of CL [108] with persistent *Leishmania* infection despite the clinical cure. In VL, persistence and subsequent relapses are particularly linked to immune-depressed patients [109], and in CanL, relapses are the rule [15]. This has been related to the *Leishmania*-containing granulomas and highlights the close connection between leishmanial infection and impairment of the functionality of the immune system [110,111].

## 2. Drug Discovery and Development of Antileishmanial Drugs

Drug discovery and development (DDD) of antileishmanial drugs follow the same pattern as any other drug: identification of hits, leads, and candidates from natural sources, chemical libraries from pharmaceutical companies or those for public access, or small chemical collections from academia. Selection includes a range of activities including toxicity tests (ADME tox), pharmaceutical chemistry and improvement of “druggability” of the molecule, vehicles to increase absorption, bioavailability, targeted distribution, excretion, and other pharmacokinetic parameters. Alternatively, when dealing with “repurposed molecules”—a cheaper approach in terms of the human labor and resources invested—the process can be considerably shortened. The process is long, expensive, and, despite all preclinical research carried out, in no way guarantees that the selected candidate will not fail at the clinical stage.

The pharma industry has been very prone to incorporate new scientific and technical approaches. In turn, this industry almost abandoned other productive sources of drugs in the past (e.g., natural sources), with the aim of reducing the high attrition rates (i.e., scarce translation from preclinical to clinical candidates) and accelerating DDD, thus reducing costs of producing new drugs. “Innovation waves” included the use of molecular biology methods (e.g., genomics, proteomics, transcriptomics, and other –omics) and selection of hits/leads/candidates based on the target-oriented and MoA paradigms [116]. This framework, combined with powerful screening robotized devices (high throughput systems (HTSs)), was expected to take us to the dawn of fast and efficient selection in the DDD process (antiparasitic drugs, specifically) [117,118]. However, HTSs have major limitations when using whole organisms (e.g., intracellular phases of *Leishmania* or *Trypanosoma cruzi*) [119] and, therefore, despite their attractive potential, to date, the combination of HTS and target-based selection has neither rendered the expectable hits and leads nor reduced the high attrition rates of DDD in any therapeutic field, including antileishmanial treatments [120,121,122,123,124].

The decline in innovation (Eroom’s law) [122,125] is factual and not restricted to antileishmanial or antiparasitic drugs but rather a general situation in DDD. As such, this is a matter of great concern for pharma companies but also for health authorities, medical doctors, veterinarians, and basic scientists. Actually, the vast majority of drugs employed against *Leishmania* infections are repurposed treatments, not new chemical entities (NCEs). In recent decades, success has been modest, and in most cases, no further development beyond the academic results has been achieved. Very few NCE have been identified in the case of leishmaniases, and no concluding evidence has been obtained in most cases when tested under field conditions [119,126,127]. In trypanosomatids, an exception could be the proteasome inhibitors (GNF6702, GSK3494245) [128,129], although their practical use is still awaited despite further development (LXE408) [130]. The in silico and bioinformatic approaches have followed a similar fate, and information on the effect of the next innovation waves (e.g., artificial intelligence, predictive platforms [131], and automation of drug discovery [132]) is not yet abundant enough to envision their possibilities, insofar that we are still waiting for a breakthrough in antiparasitic chemotherapy [121,133].

### Declining Pattern of New Drug Launches by Pharma Companies

Causes of the observed reduction in new drug launches, considering all therapeutics fields, are complex, and several factors have been incriminated though their relative importance has not been determined. Some of them are (a) exhaustion of the so considered relatively easy achievable drugs (“low hanging fruits”) of less complex chemical synthesis and the help, in the first steps of chemotherapy, of the cumulative knowledge from traditional herbal medicine; (b) higher stringency of present requirements by regulatory agencies and inadequacy of patent protection [134,135,136,137]; (c) complete separation between R&D and financial departments within the companies [138]; (d) sufficient efficacy of currently available medicines, in turn causing the “Better than the Beatles” factor [122]; (e) tendency in the pharma sector, through merges and acquisitions (M&A), toward contraction in the number of operating companies. Industrial concentration, although possibly beneficial in economic terms [139], has probably reduced competence and barely could be considered a good culture medium for innovation [140].

Some of these factors can be also recognized albeit aggravated in the chemotherapy of leishmaniasis (and other parasitic diseases). No ideal treatment exists, and patients and researchers would be sufficiently rewarded by any drug better than those presently available. Sluggish development cannot be related to the current availability of good medicines since there still are many infections without any efficacious treatment (e.g., cryptosporidiosis) and, as reviewed above, leishmaniasis treatment has serious shortcomings.

Most of the reviews on the mechanistic basis of the scarcity of new drugs deal with medicines intended for human use and global pathologies (e.g., diabetes, cancer, cardiovascular diseases), whereas most parasitic diseases and leishmaniasis, in particular, tend to be regional and, therefore, with a tighter link to the socioeconomic characteristics of the endemic region. Therefore, the lack of significant revenues for the pharma industry from antileishmanial drugs (and others against neglected tropical diseases (NTDs) (e.g., Human African Trypanosomiases, schistosomiasis, geohelminths)) has been considered one of the major factors responsible for the low investment by pharma companies and, therefore, the absence of NCE [134,141]. This consideration seems expectable from the perspective of a profit-oriented organization. However, the NCE shortage also affects parasitic diseases prevalent in developed areas, particularly in the animal health sector [142], where the revenues are significant.

## 3. Deconstructing the Drug Pipeline: Immediate Benefits but at a High Final Cost

Deconstruction and externalization of tasks (e.g., initial exploration of molecules; animal experimentation through contract research organizations (CROs)) along the drug pipeline by pharma companies probably is a reasonable strategy to reduce their operating costs and increase revenues. However, it has not allowed a significant increase in pharmaceutical storage to treat leishmaniasis or other common parasitic pathologies affecting humans and their domestic animals. On the other hand, public research institutions and universities have a high number of scientists with a chronic shortage of sufficient funds. This scenario, and the need of making drug R&D a sustainable activity [143,144], favor the setting of public–private partnerships (PPPs). There are some reputed examples in this area involving academia, public institutions, and charities (e.g., Max Planck Institute, MRC, The Carter Center, Bill and Melinda Gates Foundation) and industry (Welcome Trust, DND*i*, GSK) trying to improve the numbers in translational medicine [145]. However, effective progress has been relatively scarce, as ascertained by the current status of chemotherapy of leishmaniasis [146]. Thus, the question remains: why are there so few drugs to treat leishmaniasis (and other NTDs) despite an eager pharma sector and when so many competent scientists are involved in the identification of NCEs of potential value?

### Maybe Some Self-Criticism by Academics Is Needed

We believe it is rather unproductive trying to identify culprits in the current scarcity of new drugs since both, academia and industry, are investing their best possible resources. Thus, our goal is to pinpoint several factors that could have a higher-than-accepted impact on the efficiency of antileishmanial drugs R&D. They refer to the aim and type of research carried out in academia, and how it could act as bottlenecks of DDD and, therefore, have a direct effect on its attrition rate. In our view, these aspects are relevant, particularly when the major causes of failure are lack of effectiveness and poor safety profiles that were not predicted in preclinical and animal studies [145], or rather, in the pipeline segment where the participation of academia is more frequent. In addition, the low reproducibility of a large fraction of the research carried out [147] could lead to inefficient work and fund investments.

Research groups in academia, limited by size, funding, expertise, and publication trends, rarely can cover more than a few steps of preclinical research in the drug pipeline: synthesis of molecules, in vitro efficacy, in vitro and ex vivo MoA, toxicity for mammalian cells, and pharmacology and antileishmanial efficacy in surrogate laboratory models. Without attempting to perform a scientometric analysis, searching the term “antileishmanial” yielded over 110 publications during 2021 in PubMed on 6 October 2021. However, most of the papers are focused on a more basic exploration of potential antileishmanial molecules, e.g., design and synthesis, molecular docking, antileishmanial activity in vitro and ex vivo (59.49%; doubling the in vivo trials: 25.42%). Despite the lower predictive value of promastigotes [36], over 20% of the publications only use the antiproliferative effect on this stage to suggest the interest of the molecule or chemical family as an antileishmanial agent. Previous years probably would yield a comparable picture, and it is not uncommon that research groups use the same approach over the years without further steps toward in vivo testing.

Fragmentation of research steps between academia and industry, the standard in PPPs, is not necessarily a drawback by itself, although, frequently, the objectives of both partners are not the same. Scientists working at public institutions are aware of the limitations of this approach to DDD supported by the driving forces of publication-oriented and funding rewards [133]. Having publication records as a requirement to receive professional promotion and funding is a well-settled practice in academia. Therefore, this safe-sided research approach tends to be trenched; this is the rule both at the national and international levels. To allocate culpability on the side of promotion or funding panels should be reconsidered since promotion/fund seekers become panel members after a few years or become key opinion leaders (KOLs).

Both academia and industry share a comparable strategy for risk prevention, so do those making decisions to fund research in the public sector and development in the industry. Consequently, DDD turns into low-risk activities, and risky areas (e.g., non-publishable results; non-conventional approaches) tend to be avoided; thus, possible breakthroughs [133] are out of reach.

## 4. Reducing Bottlenecks in DDD: Some Proposals

Challenges in new drug discovery, mainly to avoid/reduce failure in the translation process, have been analyzed, both in general and considering the development of antiparasitic drugs. Potential causes of the slimmed drug pipeline include a range of items, from structural to operational, such as the scarce reproducibility of preclinical findings [145,147], the separation between preclinical and clinical investigation [148], the inadequacy of available animal models, lack of validated targets [118,145], preferential funding of basic knowledge [149], etc. Reviews specifically focused on the antiparasite DDD suggest several improvements for speeding up the process [118]. More relevant than raising new questions would be to facilitate the task by providing solutions [148], maybe through “lateral thinking”, thus challenging some of the established steps and procedures in DDD, particularly the translation from lab bench to bedside [145].

Clearly, there is room to improve anti-*Leishmania* DDD: validation of molecular targets, widening of the chemical space explored, increase in the predictive value of surrogate models, characterization of the effect of leishmaniasis on pharmacological properties of drugs and combinations, sustainability of the DDD process, an increase in funding and PPP, among others. From them, we want to emphasize some that could behave as driving/braking forces in the process of attaining a new antileishmanial drug: goal-oriented collaborative work, selection of adequate surrogate animal models, and reconsideration of target product profile (TPP) requirements. Obviously, there are other factors but, in our opinion, these three aspects play fundamental roles when exploring NCE and repurposed/repositioned drugs or drug combinations, beyond the laboratory, to effectively control the severity and extension of leishmaniasis.

### 4.1. Need of Collaborative Research Activity Sharing Objectives

Most of the efforts carried out in universities and research institutes tend to be kept within individual research niches. This has been successful in greatly increasing our knowledge, albeit fragmented, on *Leishmania* and leishmaniasis. However, this safe-sided strategy, although rewarding in strictly academic terms, is not efficient for the purpose of identifying new antileishmanial drugs or drug combinations. Academic policy exploiting research outcomes is not proactive enough, and many institutions lack the financial muscle, ability, and commitment to guarantee the effective transfer of knowledge to the industrial sector.

To overcome the difficulties posed by the fragmentation, there is a need for collaboration among research groups covering different steps. Collaboration should not be restricted to PPPs but rather to establish joint efforts among research groups to effectively identify hits and leads suitable for further development [145,148]. Given the complexity of DDD, to be effective, collaborative work requires complete transparency among partners, strict quality control procedures, long-term agreements, and ideally should cover all preclinical steps, among other substantial components. While the goal-oriented execution practices are the common strategy in the industrial environment, academic groups tend to focus on fast-rewarding experimentation in areas of expertise without scarce or null contact with adjacent—and necessary—areas preparing the background or future activities along the drug discovery pipeline. The academic–industrial partnership is surely the only way to catalyze an efficacious translation from bench to bedside [121,148,150]. Several initiatives have been established, and, despite difficulties, partial successes have been achieved with orphan drugs, drug combinations, and treatment schedules (e.g., DND*i*, GSK) [12,145,146]. Advancement along the pipeline of NCE against leishmanial infections has been sluggish, and probably other types of collaboration must be depicted. In the past, the small size of pharmaceutical companies, together with a clear goal-oriented objective, allowed complete communication among all participating scientists and technologists, rendering DDD more effective (e.g., Janssen). The present size of main pharma players and the reduced niche exploitation by academics require a clear leadership in PPP agreements and “shuttle knowledge” (i.e., forward and backward expertise) of all actors of the DDD process, as well as an optimum size of research groups [151] with a tight contact between preclinical specialists (chemists, medicinal chemists, biologists, animal scientists) and also between them and physicians engaged in clinical trials.

### 4.2. Animal Models: They Are Necessary but Not All Violins Are Stradivarius

Our predominant view has surely been, and in many cases still is, extremely reductionist considering a continuum from molecular biology to pharmacology and therapeutics [118,145,147]. Regardless of the final recipient of a drug, humans or domestic animals, experimentation is required. Up to 40 reasons for the need for animals in biomedical research have been reported by the European Animal Research Organization (EARA) (www.eara.eu; accessed on 26 November 2021). In fact, the vast majority of available antiparasitic drugs were developed through assays carried out in animal models [152]. No non-living model is predictive enough, and surrogate animal models are employed in preclinical research, both by regulatory obligation and scientific necessity. In general, attrition rates of antiparasitic agents in phase II in human patients tend to be lower, and the animal models are more predictive and less costly than those available for other pathologies [146]. This aspect is obviously more evident in the DDD of antiparasitic drugs for veterinary use. However, there is no ideal model for antiparasitic drugs intended for humans. There are exceptions such as pathologies shared by humans and domestic animals (e.g., canine leishmaniasis and human ZVL by *L. infantum* in which both mammals are natural hosts).

Availability of an animal model of infection in no way guarantees translatable results. Specific physiological and biochemical differences between hosts can limit the value of the results obtained in a surrogate species. This aspect is frequently overlooked despite the results, sometimes fatal, obtained in phase I after extensive assays in animal models (e.g., fialuridine; BIA 10-2474) [153,154,155]. Moreover, there are numerous examples of differential toxicity among species (e.g., the toxicity of penicillin for guinea pigs and hamsters but not to mice and most mammals, including humans, related to their predominant Gram-positive intestinal flora) [156], thus requiring a fine and cautious selection to develop appropriate experimental models [157].

In the case of leishmaniasis, several contributions have shown the deficiencies and possible solutions [28,38,117,158,159] and the need for appropriate animal models of VL, CL, MCL, and PKDL (i.e., mimicking infection course in the natural hosts) to reduce animal experimentation with non-human primates. Leishmanial infection elicits a strong immune response in the infected host, which leads to the resolution of the process or the extension of the disease [110,111,160,161,162,163,164]. Information on the fine-tuning of the immune system is yet fragmentary, and the laboratory models, particularly mice [165,166], are not adequate, since the Th1/Th2 dichotomy apparently is not found in any of the actual hosts of VL, humans, and dogs [167,168,169,170,171] and is surely an oversimplification [172,173].

For those scientists involved in the search for potential antileishmanial agents, drug delivery systems, or drug combinations, crossing from in vitro screening to an exploratory in vivo test in a surrogate model is not an easy task and should not be considered a straightforward step [174]. Selectivity index (SI) in phenotypic experiments using macrophages or, more frequently, monocytic cell lines (e.g., J774, THP-1), in no way precludes the possible adverse effects in the animal model employed (mice, hamster). As a rule, most research carried out and published lack a preliminary evaluation of ADME Tox characteristics of the molecule to be tested in vivo. Moreover, information obtained on antileishmanial efficacy with the current in vitro models (promastigotes, axenic amastigotes, and even better, intracellular amastigotes) does not allow the selection of either an adequate route of administration (PO, IV) or treatment schedule (dosage, duration). Due to both economic and ethical constraints, the use of animals should be limited as much as possible despite guaranteeing the significance of the results obtained. Determination of several pharmacological parameters by using simple approaches (e.g., snapshot method) [175] would give valuable clues to (1) select treatment schedules in experimental animals, (2) produce better results, and (3) reduce the number of experimental animals employed. However, these parameters are not evaluated since only 6 out of 30 in vivo trials on the efficacy of antileishmanial molecules or extracts included pharmacological studies in the references covered by PubMed during 2021 (see above).

### 4.3. Target Product Profile: Helping Guide or a Shot in the Foot?

Proposals/requirements of potential new drugs or combinations are collected in the target product profile (TPP). Since it covers very different aspects (posology, price, antiparasitic efficacy, clinical response, etc.), it may be useful to compare a new potential treatment with the available ones. However, its design is not easy, and the relative value of the items in the TPP could be tricky. TPP for primary VL [28,117,176] of a potential antileishmanial drug includes the absence of adverse events elicited by the chemotherapy; in other cases, the absence of need of medical supervision during treatment is stressed [146]. As appealing as it may seem, leishmaniasis and other NTDs are diseases linked to poverty [177,178]. Moreover, the health of an individual or a human community is beyond the treatment of a single disease and medical supervision by trained doctors would improve the general welfare.

Similarly, oral administration is favored, or it is the only one considered [117]. We wonder if this focus on oral treatment, not needing medical supervision, is related to an economic-driven strategy and the inability of organizations (NGOs, charities) and governments to change the present status more than to the actual benefits provided by this approach (higher compliance, no hospitalization).

In addition, other items considered in most TPP of antileishmanial medicines are simply overconfident. When faced with a potentially fatal disease, should a potential drug be excluded if it offers for instance a 90% efficacy, with slight adverse effects, administered for 30 days? It is doubtful that the TPPs, as written, are useful guides, particularly when in many cases they are elaborated by scientists with scarce or null connection to the clinical management of leishmaniasis in either the human or veterinary arenas. To stop the further development of a potential drug by its lack of adjustment to an unrealistic scenario (TPPs) by reducing the allocation of public or private funds would be, at least, irresponsible. Therefore, we believe that it would be convenient to reconsider the value of the TPPs published by establishing their purpose and the relevance of the items considered by including realistic objectives, in collaboration with health professionals actually performing clinical practice, in aspects such as the maximal duration of the treatment, or the “efficacy against all species and variants”, among other aspects. Otherwise, TPP could be considered a well-intentioned effort but scarcely useful for DDD.

## 5. Several Actions Could Be Taken

From the above-described situation on DDD for leishmaniasis (shared with the present shortage of new drugs in all therapeutic fields), we believe that some actions could be taken to reduce the high attrition rate of the process by increasing reproducibility and predictive value of preclinical studies, thus avoiding or minimizing the non-anticipated lack of efficacy and poor safety profile of putative NCE, repurposed drugs or drug combinations. Actions suggested are based on published seminal studies dealing with the general crisis of innovation in DDD and the specific shortage of antileishmanial medicines [34,118,120,123,144,145,146,147,148,149,158,179,180,181,182,183,184], as well as on our view after testing potential new drugs in vitro, ex vivo, and in vivo, with surrogate models and in natural hosts.

Proposals are intended to reduce, refine, and replace (3Rs) animal experimentation, when possible, and to facilitate the effective selection of potential preclinical candidates avoiding cul-de-sac, publication noise, misleading research lines, biased publication and funding, and waste of money and human resources. Some of the items considered are overlapping, thus reflecting the need for the goal-oriented collaborative nature of DDD and the need for “shuttle scientists” sliding through the pipeline. Surely some of them can be easily accomplished (e.g., more stringent in vitro and ex vivo inhibition values), whereas others need support from the scientific community (e.g., restriction of publication unless some evidence in more than one model (in vitro and in vivo) is obtained; transparency in the experimentation carried out), the commitment of scientific panels responsible of research funding by public institutions, and through PPP. Actions are primarily addressed to VL, although they could be extended to other leishmaniases and other parasitic diseases. Despite the almost evident need for some of these proposals, they are not followed in most publications.

### Proposed Actions to Select Preclinical Candidates for Treatment of Visceral Leishmaniasis

1. Validation of mean throughput systems (MHSs) and HTSs by means of a revision of the targets and parasite stages: HTSs should include the relevant parasitic stage (i.e., intracellular amastigotes) but also a validated molecular or pathway target. A combination of automatic and phenotypic screening could probably reduce the selection of irrelevant targets.
2. Use of more stringent EC values for in vitro and ex vivo tests: Use of EC90 instead for EC50, to evaluate the anti-Leishmania activity of a molecule, should drastically reduce the number of potential hits to be tested afterward. A combination of this higher stringency with a transparent and validated experimental design and methods (e.g., axenic amastigotes vs. intracellular amastigotes; culture method; adequate infection rates and culture timing) would allow for more predictive checkpoints.
3. High contents evaluation of safety and toxicity in early phases of DDD in adequate phenotypic models: In addition to the early evaluation of the selectivity index with the preferred model ex vivo (intracellular amastigotes) of a molecule, toxicity in vivo (e.g., maximum tolerated dose) in standard laboratory models (rats) must be performed before testing the efficacy in vivo.
4. Early pharmacological (PK/PD) characterization of molecules, with different administration routes, in standard animal models and, even better, in an advanced preclinical species: In vivo testing in surrogate laboratory models (mice, hamster) and non-rodents (e.g., dogs) must be preceded by, at least, a preliminary pharmacological characterization (e.g., snapshot method) to determine major pharmacological parameters (AUC, availability, half-life, excretion rate, biodistribution of the molecule). Testing in vivo a potential antileishmanial molecule without this information, besides the risk, has no scientific or ethical justification. Collaboration with medicinal chemists and pharmacists would improve the selection of the optimal presentation and administration route of the selected molecule.
5. High content evaluation in vivo of safety and efficacy: Based on scientific evidence and ethical requirements both in vivo preclinical trials performed in surrogate or advanced models should follow the 3R principles with direct participation of specialists in animal science (e.g., veterinarians) with expertise in pathophysiology of animal models (rodents, non-rodents). This is particularly evident in leishmaniasis since dogs, besides being a preclinical model for human VL, are also a natural host for ZVL, and nowadays the same drugs are used to treat VL and ZVL.
6. Transparency of animal trials including both etiological treatment and supportive therapy: Needless to say, treating a human or animal disease, e.g., leishmaniasis, is to administer an antileishmanial drug as well as to restore the physiological normality of the patient. Physicians and veterinary doctors include, in the management of any clinical case of leishmaniasis, etiological treatment against Leishmania and also supportive therapy and management indications depending on the severity of the infection, physiology impairment, both specific and unspecific immune response, hematological and biochemical deviations from normality, etc. Supportive therapy, in most instances, varies from patient to patient. However, most published reports on the evaluation of candidate antileishmanial molecules in dogs—natural host for ZVL and almost ideal preclinical model for treatment of human VL—even in studies on the comparative efficacy of marketed drugs, do not include a single mention to the administration of any medicine or individual managerial practice besides the antileishmanial drugs under consideration.
  This lack of transparency in animal experimentation is a strong drawback for any evaluation/comparison of candidate drugs and reduces the actual value of the research carried out. Eventually, the probably different and unreported supportive therapy administered could be responsible for the sometimes-disparate results obtained in different studies with the same drug. The situation is not substantially better in clinical trials. Unless complete and transparent information is provided, such studies probably lack significance.
7. Network design and flexible structure of DDD process: With the fragmentation of the DDD process (e.g., externalization to CRO, collaborative work through PPPs), the canonical scheme of drugs’ pipeline in the preclinical steps should be reconsidered. Networking rather than a lineal pipeline with a clear goal-oriented leadership could reduce the go/no-go decision checkpoints, thus accelerating the preclinical process. Open access to data repositories by partners will ensure a more precise assessment of the actual value of the results, possible hurdles, and solutions to progress.

Pharma companies look for revenues to cover the amounts invested in both personnel and equipment (e.g., robotics for HTS). This understandable policy by private industry, together with the general academic activity and funding agencies, favors the conservatism of the prevailing antileishmanial DDD model despite the limited success achieved. Investment by both academia and industry has been considerable, sometimes with disparate calculations [185]. Surely, after more than 30 years with insignificant or very limited success, it is time to reevaluate the actual contribution of the prevailing practices in DDD, and, eventually, abandon them if they reveal unfruitful.

## Figures and Tables

**Table 1:** Antileishmanial drugs currently used against visceral (VL), cutaneous (CL), mucocutaneous (MCL), and post-kala-azar dermal leishmaniasis (PKDL): indication (X) and year of introduction in the market.

Drug [Reference]	VL	CL	MCL	PKDL	Introduction Year
Pentavalent antimonials [41,54,112]	X	X	X	X	1937–1945
Amphotericin B [41,77,112]	X			X	1959
Miltefosine [41,113]	X	X	X		2002
Paromomycin [100]	X	X			2006
Pentamidine [41,114]	X	X	X		1973
Azoles [115]		X	X		1980s
Allopurinol [113]	X				1980s
